# Investigation of antimicrobial and antioxidant activities of *Chenopodium album* extracts and their effects on gentamicin nephrotoxicity in rats

**DOI:** 10.1002/fsn3.3733

**Published:** 2023-10-05

**Authors:** Perihan Akbaş, Elife Kaya, Mustafa Makav, Gülden Yıldız

**Affiliations:** ^1^ Karadeniz Advanced Technology Research and Application Center Ondokuz Mayıs University Samsun Turkey; ^2^ Department of Food Processing, Technical Sciences Vocational School Kahramanmaras Sutcu Imam University Kahramanmaras Turkey; ^3^ Department of Physiology, Veterinary Faculty Kafkas University Kars Turkey; ^4^ Department of Pathology, Faculty of Medicine Kafkas University Kars Turkey

**Keywords:** antimicrobial, antioxidant, *Chenopodium album*, gentamycin‐induced nephrotoxicity

## Abstract

This study aims to examine the antimicrobial and antioxidant activities of the aerial parts of *Chenopodium album* extracts (CAE) prepared with different solvents, and how *C. album* ethanol extract protects them against gentamicin‐induced nephrotoxicity. Extracts of *C. album* aerial parts were obtained from ethanol, water, methanol, chloroform, and hexane solvents. Thirty‐two male *Wistar albino* rats were used and gentamycin‐induced nephrotoxicity was utilized as a model. The water extract of *C. album* exhibited no antimicrobial effect, whereas the methanol one created the highest zone diameter on *Bacillus cereus* (26 mm). The methanol extract displayed the highest activity in DPPH and ABTS. The ethanol extract yielded the highest reducing power in the CUPRAC. The water extract had the highest reducing power in the FRAP. Concerning gentamicin‐induced renal damage, creatinine and urea levels in the blood were statistically higher in the gentamicin‐*C. album* group compared to the other groups (*p* < .05). Urea and creatinine levels of the gentamicin‐*C. album* group dropped significantly, indicating that the *C. album* was effective against renal damage. The sections from kidney tissues in the gentamicin + *C. album* group mostly exhibited mild glomerular congestion. Hyaline cast, cytoplasmic vacuolization, necrosis, and apoptosis were not observed. Thanks to *C. album* treatment, the gentamicin + *C. album* suffered less histopathological damage than the gentamicin group did. The results of the present study suggest that CAE can be used as a supportive treatment in people undergoing treatment for nephrotoxicity.

## Graphical abstract

The therapeutic effect of *Chenopodium album* against nephrotoxicity was determined for the first time in this study. It was observed that ethanol extract of *C. album* significantly decreased the levels of urea and creatinine in gentamicin‐induced kidney damage. Among the extracts of *C. album* prepared with different solvents, polar solvents showed better antioxidant activity. It was determined that the water extract of *C. album* did not have antimicrobial activity, but other extracts had a strong antimicrobial effect.

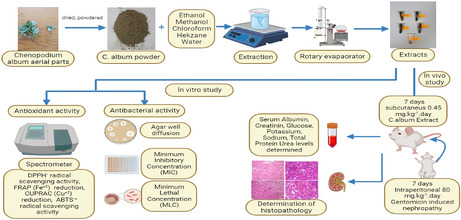

## 1 INTRODUCTION

Leafy green vegetables have attracted people's interest across the world due to their many health benefits. They have been used for medicinal purposes since antiquity. Plant‐based medications are increasingly taking the place of synthetic drugs, which are routinely used in the treatment of ailments. Throughout the history of humanity, knowledge about the reason behind consuming different plants, which ones are toxic, and which ones have medicinal properties has been gained. Likewise, medicinal and aromatic plants have been used for their antibacterial and antioxidant properties, as well (Lone et al., 2017; Pourghorban et al., 2022). Many microorganisms have developed multi‐drug resistance because of misused medicine. Many studies have investigated antimicrobial compounds derived from natural sources with the aim of elucidate their properties and potential applications. In comparison of plants and synthetic pharmaceuticals, it is evident that the former are more accessible and affordable (Arslan & Kaya, 2021).

The plant *Chenopodium album* (Chenopodiaceae) is distributed all around the world and is a member of the genus *Chenopodium*, which includes about 250 species. It is also known in English as “white goosefoot” or “wormseed,” and can be annual, biennial, or perennial in nature. Its medicinal properties mainly hail from its leaves and seeds (Sikarwar et al., 2017), for which it is well known (Choudhary & Sharma, 2014). It is rich in protein, fiber, minerals, vitamins, and vital fatty acids. By extension, *C. album* has many pharmacological properties, and has been traditionally used to treat peptic ulcers, indigestion, flatulence, sore throat, pharyngoplasty, splenopathy, ophthalmopathy, and general malaise (Poonia & Upadhayay, 2015). Current data from both in vitro and in vivo studies indicate that it has many antiviral, anti‐inflammatory, anti‐allergic, antiseptic, antipruritic, and antinociceptive properties, as well as an immunomodulatory effect against sperm immobilization (Kaur & Kapoor, 2002; Kumar et al., 2007). Similarly, it has antispasmodic (García et al., 1997), antiparasitic (Giove Nakazawa, 1996), antimicrobial, antifungal (Maksimović et al., 2005), and antioxidant properties and is beneficial for people who suffer from peptic ulcers and heart disease (Laghari et al., 2011; Mousavi et al., 2005; Poonia & Upadhayay, 2015; Saini et al., 2019). *Chenopodium album* leaves are used to treat kidney and urinary tract disorders, to purify blood, to protect people from contracting roundworms and hookworms (anthelmintic), to induce laxation, and to treat liver diseases, splenomegaly, burns, and intestinal ulcers (Sarma et al., 2008).

The generation of free oxygen radicals in the renal cortex is a key aspect in the pathology of gentamicin (GM) nephrotoxicity. Nephrotoxicity is one of the most common causes of acute kidney injury. Numerous treatments prevent gentamicin‐induced nephrotoxicity, and many free radical scavengers have been scientifically shown to reduce the effect of gentamicin, and ultimately improve nephrotoxicity (Walker & Shah, 1988).

Polyphenols are the most important component of *C. album*, and responsible for several of its pharmacological activities. Polyphenols, namely flavonoid derivatives, function as reducing agents, and effectively quench singlet oxygen giving them hydrogen (Bai et al., 2023; Poonia & Upadhayay, 2015). Plants contain phenolic compounds. They act as reservoirs for antibacterial and antioxidant substances, hence showing their potential medicinal properties. The redox properties of phenolic compounds allow them to serve as hydrogen donors, reducing agents, and singlet oxygen quenchers, thus turning them into antioxidants. They are also shown to be potent antioxidants capable of stopping or slowing the pace of oxidation, a free radical chain reaction that occurs in materials that are susceptible to oxidation (Tarnawski et al., 2006; Wang et al., 2022). The flavonoid content in *C. album* species and their associated biological activities have been studied by Vysochina (2010). The most important flavonoids contained in *C. album* consist of 3‐O‐glycosides of kaempferol, quercetin, and isorhamnetin. *Chenopodium album* also contains phenolic amide (Hirano et al., 2001). Several compounds including apocarotenoids (DellaGreca et al., 2004), saponin (Lavaud et al., 2000), alkaloid chenoalbicin, and cinnamic acid amide, alongside phenols and lignans (Cutillo et al., 2006), have been identified. Laghari et al. (2011) discovered that *C. album* contains seven phenolic acids, including vanillin, vanillic acid, protocatechuic acid, caffeic acid, syringic acid, protocatechuic aldehyde, and gallic acid. The phenolic acids in *C. album* are common (Liu et al., 2023). Its anti‐oxidative effect is mostly associated with these phenolic components such as flavonoids (Pietta, 2000), phenolic diterpenes, and phenolic acids (Naczk & Shahidi, 1992).

This study aims to investigate the antimicrobial and antioxidant activities of *C. album* as well as how the ethanol extract of the plant could protect rats against gentamicin‐induced nephrotoxicity. As no studies to date have focused on nephrotoxicity effect of the *C. album* plant, this study is a pioneer in this regard.

## 2 MATERIALS AND METHODS

### 2.1 Materials

#### 2.1.1 Chemicals

All of the solvents for extraction and other chemicals used were of analytical grade and provided by Sigma (Sigma‐Aldrich GmbH). Mueller Hinton agar and broth were obtained from Merck.

#### 2.1.2 Microorganisms

The standard strains of *Bacillus subtilis* (ATCC 6633), *Staphylococcus aureus* (ATCC 25213), *Klebsiella pneumoniae* (ATCC 13883), *Yersinia enterocolitica* (ATCC 9610), *Bacillus cereus* (ATCC 14579), *Escherichia coli* (ATCC 25922), *Pseudomonas aeruginosa* (ATCC 15442), and *Pasteurella multocida* (ATCC 12945) were all used to analyze the antimicrobial effects of the extracts.

### 2.2 Method

#### 2.2.1 Preparation of the plant extracts

First, the plant was harvested from Kümbetli village, Kars in Turkey (40.54736, 43.0095) in May 2021. *Chenopodium album* subsp. *album* were identified by Professor Dr. Hasan KORKMAZ, Ondokuz Mayıs University, Biology Department, Samsun. A specimen (OMU‐8910) was preserved in the herbarium at the Biology Department. Then, its species was verified on a plant database (http://www.theplantlist.org/). Afterward, its aerial parts were ground into a fine powder. Ten grams of plant was then placed in 200 mL of water, ethanol, methanol, chloroform, and hexane (each with different polarities) at a ratio of 1:20 for solvents. Lastly, the extracts were filtered, the solvents were evaporated, and the water extract was lyophilized.

#### 2.2.2 Preparing the bacterial cultures, and identifying the minimum inhibition concentration and minimum lethal concentration using agar well diffusion

First, 1 mg of each of the powdered extracts were prepared for antimicrobial activity testing by adding them to 1 mL of dimethyl sulfoxide (DMSO), an organic solvent. The agar well diffusion method (Akbaş et al., 2020) was applied to detect the plant's antimicrobial activity. After the bacterial suspension was adjusted according to the 0.5 McFarland standard, it was inoculated in Mueller Hinton Agar Petri dishes. Next, 5‐mm‐diameter wells were drilled on the agar, upon which 50 μL (50 μg) of the extracts were transferred into them. Then, they were left to incubate at 37°C for 24 h, and then their zone diameters were measured. Ampicillin (20 μg/well) was administered as a control. In vitro, antimicrobial activity was tested by determining the minimum inhibitory concentration (MIC) and minimum lethal concentration (MLC) using micro‐dilution method (Khomarlou et al., 2017).

#### 2.2.3 Radical scavenging and reducing assays

The DPPH and ABTS radical scavenging activities of the extracts (10–80 μg mL^−1^) were calculated using methods from three separate studies (Arslan & Kaya, 2021; Blois, 1958; Re et al., 1999). CUPRAC assay is a chromogenic redox reaction. FRAP method is based on reduction of the Fe^3+^‐TPTZ complex under acidic conditions. For reduction activities, CUPRAC and Fe^2+^‐TPTZ were spectrometrically reported at 450 and 593 nm as described in the study (Apak et al., 2004; Sehitoglu et al., 2015). Ultimately BHA and α‐Tocopherol were chosen to serve as the standard.

#### 2.2.4 Nephrotoxicity assay

To conduct this study, approval was first obtained from the Experimental Animal Research Ethics Committee of Kafkas University (no: KAUHADYEK 2021/12). Thirty‐two male *Wistar albino* rats (2–3 months of age, 250–350 g) – 8 rats per group – were used as subjects. All of the procedures were carried out according to European Community guidelines (EEC Directive of 1986; 86/609/EEC) regarding how to use and care for laboratory animals. Those in the experimental groups were injected with 80 mg kg^−1^ of gentamicin intraperitoneally once a day to induce acute renal injury (Akyüz et al., 2021). The following groups were formed to investigate what effects *C. album* ethyl alcohol extract had (0.45 mg kg^−1^) on nephrotoxicity.

1. Control Group (C) (*n*: 8): Healthy rats, medication free (they were given oral physiological saline solution instead).
2. *C. album* Group (CA) (*n*: 8): Injected with a single dose of 0.45 mg kg^−1^ of *C. album* extract, subcutaneously.
3. Gentamicin group (G) (*n*: 8): Injected every day with a single dose of 80 mg kg^−1^ of gentamicin intraperitoneally.
4. Gentamicin *C. album* Group (G + CA) (*n*: 8): Injected every day with a single dose of 80 mg kg^−1^ of gentamicin, intraperitoneally, as well as with a single dose of 0.45 mg kg^−1^ of *C. album* extract subcutaneously.

All the rats were euthanized by cervical dislocation under anesthesia (ketamine hydrochloride [75 mg kg^−1^] and xylazine [15 mg kg^−1^], intramuscularly) at the end of the study, and blood and tissue samples were collected from them. The blood samples were stored at −20°C for further analysis. The tissue samples were stored in 10% buffered formaldehyde for histopathological analysis.

#### 2.2.5 Biochemical method

Glomerular filtration rate (GFR) was measured by Bromo cresol purple; urea by Urease UV, creatinine (CREA) by alkaline picrate‐kinetic rate blanked, total protein (TP) by Biuret endpoint, glucose 6‐phosphate (GLU) by hexokinase, sodium (Na), and potassium (K) by ion‐selective electrode method.

#### 2.2.6 Histopathological method

The tissues were fixed in 10% buffered formaldehyde for 8 h. After normal histological follow‐up, they were embedded in paraffin, then cut into 4 μm strips, and stained with hematoxylin and eosin. Finally, histopathological features were studied under a light microscope (Olympus BX46).

#### 2.2.7 Statistical analysis

Antimicrobial and antioxidant activity assays and biochemical analyses were done with at least three replicates. The data were analyzed using One‐Way Analysis of Variance (one‐way ANOVA). The values of *p* < .05 were deemed statistically significant. Statistical analyses were carried out on SPSS (version 20).

## 3 RESULTS

### 3.1 Antimicrobial activity results

Table 1 shows the antimicrobial activity results of the aerial parts of *C. album* as used for the different extracts. Table 2 shows MIC and MLC concentrations of the bacteria.

**TABLE 1:** Inhibition zone diameters of the aerial parts of *Chenopodium album* on the test bacteria.

Bacteria/extracts	Water (50 μg)	Ethanol (50 μg)	Methanol (50 μg)	Chloroform (50 μg)	Hexane (50 μg)	Ampicillin (20 μg)
*Staphylococcus aureus*	R	15.6 ± 0.5^e^	20.7 ± 0.7^c^	20.3 ± 0.8^c^	20.4 ± 0.9^c^	22.3 ± 0.6^b^
*Bacillus subtilis*	R	12.2 ± 0.7^g^	20.3 ± 0.9^c^	18.3 ± 1.1^d^	20.3 ± 0.7^c^	27.2 ± 0.6^a^
*Klebsiella pneumoniae*	R	14.6 ± 0.4^f^	22.2 ± 0.8^b^	12.2 ± 0.3^g^	R	20.2 ± 0.8^b^
*Bacillus cereus*	R	14.1 ± 1.1^f^	26.0 ± 1.0^a^	20.9 ± 0.2^c^	14.3 ± 0.7^f^	26.8 ± 0.8^a^
*Pseudomonas aeruginosa*	R	20.4 ± 0.5^c^	24.5 ± 0.8^ab^	18.2 ± 0.6^d^	18.4 ± 0.5^d^	R
*Escherichia coli*	R	20.7 ± 0.8^c^	24.3 ± 0.8^ab^	14.2 ± 0.9^f^	12.3 ± 0.8^g^	22.2 ± 0.7^b^
*Yersinia enterocolitica*	R	R	20.5 ± 0.6^c^	16.3 ± 0.3^e^	R	10.4 ± 0.8^h^
*Pasteurella multocida*	R	R	16.9 ± 0.7^e^	R	R	16.4 ± 0.4^e^

*Note*: The figures that are represented in different letters in the same row and column are different at the level of *p* < .05.

Abbreviation: R, Resistant.

**TABLE 2:** MIC and MLC values of the extracts (μg mL^−1^).

Bacteria/extracts	Water		Ethanol		Methanol		Chloroform		Hexane		Ampicillin	
	MIC	MLC	MIC	MLC	MIC	MLC	MIC	MLC	MIC	MLC	MIC	MLC
*Staphylococcus aureus*	R	R	500	500	62.5	125	125	250	250	250	250	250
*Bacillus subtilis*	R	R	500	500	31.75	31.75	125	125	250	500	62.5	125
*Klebsiella pneumoniae*	R	R	500	500	62.5	62.5	R	R	R	R	62.5	125
*Bacillus cereus*	R	R	500	500	62.5	125	125	250	250	250	31.75	62.5
*Pseudomonas aeruginosa*	R	R	250	500	62.5	125	125	125	250	250	R	R
*Escherichia coli*	R	R	250	500	62.5	125	500	500	R	R	62.5	62.5
*Yersinia enterocolitica*	R	R	R	R	16	16	500	500	R	R	500	500
*Pasteurella multocida*	R	R	R	R	31.75	31.75	R	R	R	R	250	250

*Note*: The figures that are represented in different letters in the same column are different at the level of *p* < .05.

Abbreviation: R, Resistant.

The methanol extract formed the largest zone diameter on *B. cereus* (26 mm). Moreover, the ethanol, methanol, chloroform, and hexane extracts exhibited antimicrobial effects on all test microorganisms. The water extract had no antimicrobial activity (Tables 1 and 2).

As indicated in Table 1, the methanol extract of *C. album* exhibited the largest zone diameter (26 mm) against *B. cereus*. The methanol extract of *C. album* has greater antibacterial efficacy compared to Ampicillin (20 μg) against *P. aeruginosa* (24 mm), *E. coli* (24 mm), and *Y. enterocolitica* (20 mm). The zone diameter that had formed from the methanol, chloroform, and hexane extracts on *S. aureus* (20 mm) was smaller compared to the zone diameter created by Ampicillin (20 μg) (22 mm), but was still significantly greater (Table 1).

Based on Table 2, the values of MIC and MLK were compatible with zone diameters. The lowest MIC value for *Y. enterocolitica* appeared in the methanol sample with 16 μg mL^−1^. At 31.75 μg mL^−1^ of the methanol extract, both *B. subtilis* and *P. multocida* had stopped growing. As for the MIC and MLC levels, the methanol extract proved itself to be the effective against test bacteria, whereas its water extract showed no antimicrobial activity on test bacteria. Ethanol and hexane extracts demonstrated no antimicrobial activity against *Y. enterocolitica* and *P. multocida*, whereas *P. multocida* was resistant to chloroform extract and *K. pneumoniae* was resistant to hexane extract.

### 3.2 Antioxidant activity results

Table 3 shows the antioxidant activity results of *C. album* extracts that were made in water, ethanol, methanol, chloroform and hexane solvents with different polarities, and standard antioxidants at different concentrations.

**TABLE 3:** The half maximal inhibitory concentration (IC_50_ μg mL^−1^) for the DPPH/ABTS radical scavenging activities, and CUPRAC/FRAP (80 μg mL^−1^) reducing power results of the extracts from *Chenopodium album* and standards.

Antioxidants	DPPH^•^ scavenging^1^	ABTS^•+^ scavenging^1^	Cu^2+^ reducing (CUPRAC)^2^	Fe^3+^‐TPTZ reducing (FRAP)^2^
Water	86.27 ± 0.27^e^	59.62 ± 0.05^d^	0.47 ± 0.01^d^	0.30 ± 0.00^c^
Ethanol	75.49 ± 0.03^d^	63.28 ± 0.09^e^	0.53 ± 0.00^c^	0.26 ± 0.00^d^
Methanol	55.42 ± 0.05^c^	36.91 ± 0.13^c^	0.31 ± 0.01^e^	0.24 ± 0.00^e^
Chloroform	102.24 ± 0.16^f^	95.21 ± 0.05^f^	0.28 ± 0.02^f^	0.18 ± 0.00^f^
Hexane	152.53 ± 0.79^g^	110.25 ± 0.13^g^	0.19 ± 0.00^g^	0.11 ± 0.00^g^
BHA	15.21 ± 0.02^b^	19.01 ± 0.04^b^	0.86 ± 0.00^b^	0.96 ± 0.00^b^
α‐Tocopherol	9.12 ± 0.03^a^	11.74 ± 0.03^a^	0.98 ± 0.00^a^	1.23 ± 0.00^a^

*Note*: Each point shows the average value of three replicates ± SD. The figures that are represented in different letters in the same column are different at the level of *p* < .05.

Abbreviations: ABTS, 2,2‐azinobis (3‐ethylbenzo‐thiazoline‐6‐sulfonic acid); BHA, butylated hydroxyanisole; DPPH, 2, 2‐diphenyl‐1‐ picrylhydrazyl radical.

^1^ Expressed as IC_50_ μg mL^−1^ values.

^2^ Expressed as absorbance values.

DPPH and ABTS antioxidant activity methods were used to both detect the radical scavenging effect of the plant extracts, and to assess the ability of both lipophilic and hydrophilic antioxidants to scavenge free radicals via either hydrogen or electron transfer. The IC_50_ value is widely used for indicating antioxidant properties. A lower IC_50_ value indicates stronger antioxidant power. Therefore, IC_50_ values were also calculated to compare and contrast the ABTS/DPPH scavenging activities of all of the extracts against α‐tocopherol and BHA.

Each extract's IC_50_ value was as follows: 9.12 ± 0.03 μg mL^−1^ for α‐tocopherol, 15.21 ± 0.02 μg mL^−1^ for BHA, 55.42 ± 0.05 μg mL^−1^ for methanol, 75.49 ± 0.03 μg mL^−1^ for ethanol, 86.27 ± 0.27 μg mL^−1^ for water, 102.24 ± 0.16 μg mL^−1^ for chloroform, and 152.53 ± 0.79 μg mL^−1^ for hexane (Table 3). The DPPH scavenging activity was stronger when the IC_50_ value was lower. The order of DPPH radical scavenging powers of *C. album* extracts and standards according to their IC_50_ values was: α‐tocopherol > BHA > methanol > ethanol > water > chloroform > hexane.

Likewise, for the ABTS, the extracts had the following IC_50_ values: 11.74 ± 0.03 μg mL^−1^ for α‐tocopherol, 19.01 ± 0.04 μg mL^−1^ for BHA, 36.91 ± 0.13 μg mL^−1^ for methanol, 59.62 ± 0.05 μg mL^−1^ for water, 63.28 ± 0.09 μg mL^−1^ for ethanol, 95.21 ± 0.05 μg mL^−1^ for chloroform, and 110.25 ± 0.13 μg mL^−1^ for hexane. The ABTS^•+^ scavenging ability of *C. album* extracts and standards according to their IC_50_ values was α‐tocopherol > BHA > methanol > water > ethanol > chloroform > hexane, respectively (Table 3). A lower IC_50_ value indicates a higher level of ABTS scavenging activity. Based on this data, methanol exhibited the best radical scavenging activity in both methods.

Reducing power of the plants is essential to assess their antioxidant potential. Reducing power tests are an indicator of the ability of antioxidant compounds to donate electrons. For this purpose, the reducing power of the plant extracts was investigated by using CUPRAC and FRAP. The reduction activities of cupric ion (Cu^2+^) at 80 μg mL^−1^ were α‐tocopherol (*λ*_450_: 0.98 ± 0.00) > BHA (*λ*_450_: 0.86 ± 0.00) > ethanol (*λ*_450_: 0.53 ± 0.00) > water (*λ*_450_: 0.47 ± 0.01) > methanol (*λ*_450_: 0.31 ± 0.01) > chloroform (*λ*_450_: 0.28 ± 0.02) > hexane (*λ*_450_: 0.19 ± 0.00). In FRAP test, it was α‐tocopherol (*λ*_593_: 1.23 ± 0.00) > BHA (*λ*_593_: 0.96 ± 0.00) > water (*λ*_593_: 0.30 ± 0.00) > ethanol (*λ*_593_: 0.26 ± 0.00) > methanol (*λ*_593_: 0.24 ± 0.00) > chloroform (*λ*_593_: 0.18 ± 0.00) > hexane (*λ*_593_: 0.11 ± 0.00). The high absorbance in this method indicates high antioxidant capacity (Table 3).

### 3.3 Results of the nephrotoxicity assay

**FIGURE 1:**
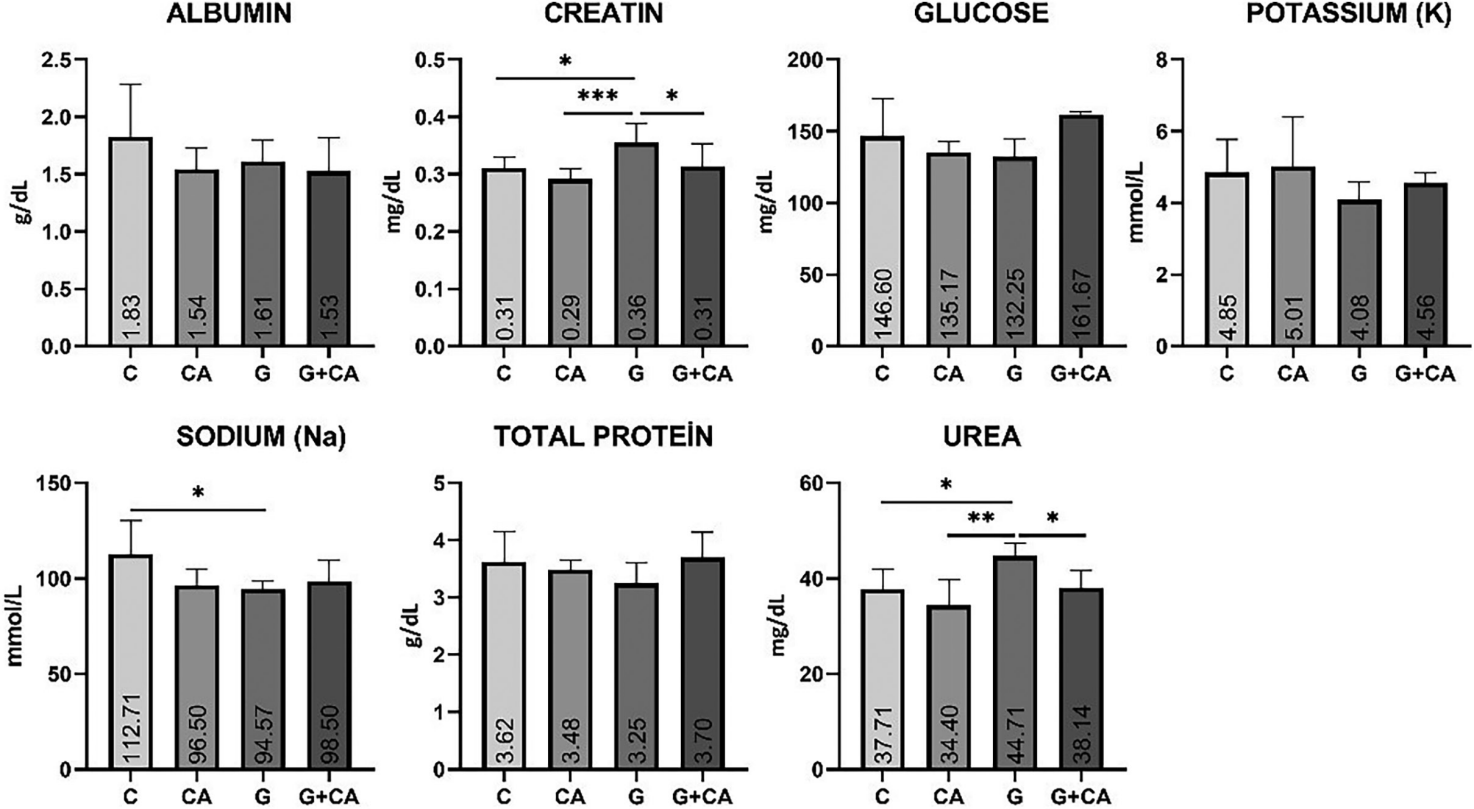
Albumin, creatinine, glucose, potassium, sodium, total protein, and urea levels in blood. Means and SD of the four groups for biochemical parameters. *p < .05; **p < .01; ***p < .001.

Some biochemical parameters were examined to determine whether any of the rats suffered renal damage. Figure 1 shows albumin, creatinine, glucose, potassium, sodium, total protein, and urea levels in their blood. Creatinine and urea levels statistically significantly increased in the blood of the rats with gentamicin‐induced renal damage, when compared with the other groups (*p* < .05).

By contrast, creatinine and urea levels dropped significantly in the gentamicin‐*C. album* (G + CA) group compared to the gentamicin (G) group, indicating that *C. album* was effective against renal damage.

Na values also significantly dropped in blood in the G group compared to the control (C) group (*p* < .05). Even though there was no statistical difference between the G and G + CA groups, a quantitative increase was observed in the G + CA group. As for albumin, glucose, total protein, and potassium levels, no statistically significant difference was found between the groups (*p* < .05).

### 3.4 Histopathological findings

Lipid peroxidation leads to the breakdown of cellular membranes and subsequent shedding of the contents of the cytoplasm into the tubular lumen and can account for the appearance of hyaline casts (Taha et al., 2023).

**FIGURE 2:**
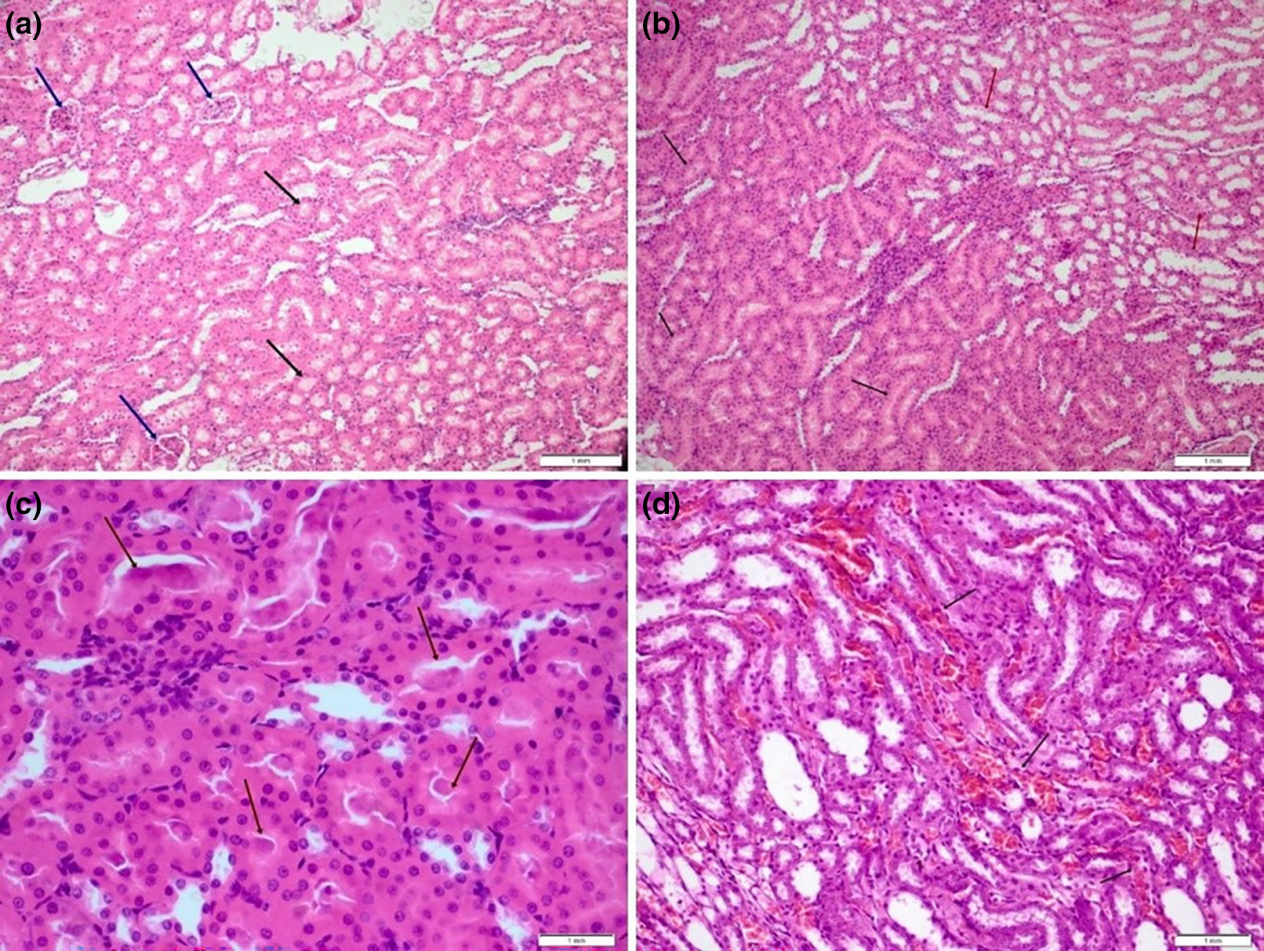
(a) section of kidney tissue from the control group. Blue arrows: glomeruli, Black arrows: tubules. (b) Kidney section from *Chenopodium album* group in ordinary histomorphology, black arrow: proximal tubules, red arrow: distal tubules. (c) Hyaline cast deposits in the tubules of kidney sections from gentamicin group (arrows). (d) Findings of moderate congestion in kidney section from gentamicin + *C. album* group (black arrows), hematoxylin and eosin (H&E) ×100.

Nephrotoxicity is directly responsible for inducing tubular damage, fibrogenesis, oxidative stress, vascular abnormalities, inflammation, necrosis, and apoptosis (Prasad et al., 2004). Consequently, an investigation was conducted on the renal impairments, including histopathological alterations such as presence of hyaline cast, glomerular congestion, cytoplasmic vacuolization, necrosis, and apoptosis. Hematoxylin eosin sections, taken from eight paraffin‐embedded renal tissues from all of the groups, were analyzed under a light microscope. Figure 2 shows images of kidney tissue sections. No cytoplasmic vacuolization, necrosis, or apoptosis were seen in any of the groups. Table 4 shows the evaluation of tissues for glomerular congestion. Hyaline cast occurred solely in the gentamicin group (Table 5), indicating that the cellular membrane in the kidneys was disrupted.

**TABLE 4:** Histopathological scoring of kidney sections (glomerular congestion) from the control (C) group, *Chenopodium album* (CA) group, gentamicin (G) group, and gentamicin + *C. album* (G + CA) group.

Glomerular congestion score	Control (C) group	*C. album* (CA) group	Gentamicin (G) group	Gentamicin + *C. album* (G + CA) group
1st Kidney section	1	1	1	1
2nd Kidney section	1	1	1	1
3rd Kidney section	1	1	1	1
4th Kidney section	1	1	1	2
5th Kidney section	2	1	1	1
6th Kidney section	2	2	1	1
7th Kidney section	1	1	1	1
8th Kidney section	1	1	1	1

*Note*: 0: No symptoms, 1: Mild symptoms, 2: Moderate symptoms, 3: Severe symptoms.

**TABLE 5:** Histopathological scoring about hyaline cast on kidney sections from the control (C) group, *Chenopodium album* (CA) group, gentamicin (G) group, and gentamicin + *C. album* (G + CA) group.

Hyaline cast	Control (C) group	*C. album* (CA) group	Gentamicin (G) group	Gentamicin + *C. album* (G + CA) group
1st Kidney section	0	0	0	0
2nd Kidney section	0	0	1	0
3rd Kidney section	0	0	0	0
4th Kidney section	0	0	0	0
5th Kidney section	0	0	1	0
6th Kidney section	0	0	2	0
7th Kidney section	0	0	0	0
8th Kidney section	0	0	0	0

*Note*: 0: No symptoms, 1: Mild symptoms, 2: Moderate symptoms, 3: Severe symptoms.

C group had mild to moderate glomerular congestion, but no hyaline cast, cytoplasmic vacuolization, necrosis, or apoptosis. Figure 2a shows section of kidney tissue from the C group.

When examining the kidney sections from the CA group in ordinary histomorphology. *Chenopodium album* group also exhibited mostly mild glomerular congestion (Table 4), again with no hyaline cast, cytoplasmic vacuolization, necrosis, or apoptosis. Figure 2b shows CA group.

Mild glomerular congestion was observed in all of the kidney samples in the G group. Mild hyaline cast was found in two samples and moderate hyaline cast was detected in one sample (Table 5). No cytoplasmic vacuolization, necrosis, nor apoptosis were discovered. Figure 2c shows hyaline cast deposits in the tubules of kidney sections from Gentamicin group.

According to Figure 2d, kidney tissue changes took place in the G + CA group. The G + CA group also had mostly mild glomerular congestion, but no hyaline cast (Tables 4 and 5).

## 4 DISCUSSION

Various physiological and biochemical processes that take place in the human body usually generate different types of reactive oxygen species and oxygen‐containing free radicals. In biological systems, the compounds that eliminate or destroy reactive oxygen species not only exist in human metabolism, but also in plants and fruits. Phytocomponents such as phenolic compounds and flavonoids – which are generally available in plants – are responsible for antioxidant properties and have nephroprotective properties. This makes medicinal plants a great potential source for pharmaceutical products. This study therefore aimed to identify the antimicrobial and antioxidant activities of *C. album*, and examine what effects they have on nephrotoxicity.

*Chenopodium album* has antimicrobial activity against different pathogenic bacteria such as *P. multocida*, *K. pneumoniae*, *Bacillus cereus*, *S. aureus* , *P. aeruginosa*, *B. subtilis*, *E. coli*, and *Y. enterocolitica* (Adedapo et al., 2011; Khomarlou et al., 2017; Külcü et al., 2019; Parkash & Patel, 2014). Korcan examined the antibacterial activity of methanolic extract and found that it was most effective against *B. subtilis* (13 mm zone of inhibition at 100 g mL^−1^), and that the activity increased with increasing the extract concentration (Korcan et al., 2013). Lone et al. (2017) observed that the methanolic extract had a maximal inhibitory action against *S. aureus* (28 mm) and a mild impact against *E. coli* (Singh et al., 2023). This study revealed that hexane, methanol, ethanol, and chloroform extracts of *C. album* had an antimicrobial effect on experimental bacteria, while water extract had no antimicrobial activity. The methanol extract of *C. album* produced the largest zone diameter (26 mm) on *B. cereus*. The antimicrobial activity of the methanol extract of *C. album* against *P. aeruginosa*, *E. coli* and *Y. enterocolitica* was quite high. For *Y. enterocolitica*, the MIC value of the methanol extract of *C. album* was 16 μg mL^−1^. *Chenopodium album* exhibited a strong potential antimicrobial effect.

The antioxidant activity capacities of plants vary according to the analytical method applied. As indicated in Table 3, hexane and chloroform extracts had low DPPH and ABTS radical scavenging capabilities, with high IC_50_ values. According to the IC_50_ values, methanol extracts (IC_50_: 55.42 ± 0.05 μg mL^−1^, IC_50_: 36.91 ± 0.13 μg mL^−1^, respectively) had a significant effect on the DPPH and ABTS radicals, but are weak radical scavengers compared to BHA (IC_50_: 15.21 ± 0.02 μg mL^−1^, IC_50_: 19.01 ± 0.04 μg mL^−1^, respectively) and α‐tocopherol (IC_50_: 9.12 ± 0.03 μg mL^−1^, IC_50_: 11.74 ± 0.03 μg mL^−1^, respectively). The related studies have revealed that methanol extract among extracts prepared with different solvents (Adedapo et al., 2011; Laghari et al., 2011) has the highest activity. When the DPPH radical scavenging activity was compared with the literature, the methanol extract's radical scavenging ability proved to be highly effective (Lone et al., 2017; Pandey & Gupta, 2014). Another study reported that *C. album* extract had comparably important free radical scavenging activity compared to standard antioxidant BHT (Islam et al., 2023). FRAP works by reducing Fe^3+^ to Fe^2+^ in an acidic medium. Fe^2+^ forms in the reaction medium and creates a colored complex structure by absorbing at 593 nm with TPTZ. The absorbance change follows the intensity of this reaction (Albayrak et al., 2010). The water extract of the *C. album* plant exhibited the highest antioxidant activity in the study conducted by the FRAP method. The study conducted with the CUPRAC method based on the reduction of Cu^2+^ to Cu^3+^ exhibited the highest antioxidant activity in the ethanol extract of the plant. The ethanol extract exhibited the highest activity in the study by Külcü et al. (2019) using the CUPRAC method. Another study using FRAP indicated that water and methanol extracts of *C. album* exhibited a good antioxidant activity (Pandey & Gupta, 2014). Based on the antioxidant activity analysis, the antioxidant activities increased and differed depending on the concentration. This difference was associated with factors such as different solvents and extraction methods used, the environment in which the plant was cultivated, and the time of harvesting. Also, solvents with various polarities were used for extraction in the study. The results of the study indicated that the polar solvents yielded better extraction efficiencies (Ahmed et al., 2014), which is compatible with the present study.

Aminoglycosides are antibiotics used to treat infections caused by Gram‐positive and Gram‐negative aerobes – either alone or in combination with active cell wall agents. Gentamicin (G) is the most widely administered antibiotic among all aminoglycosides, primarily to treat Gram‐negative bacterial infections (Ali et al., 2011; Althobaiti et al., 2023; Choi et al., 2011). However, taking it for more than 7 days can cause severe ototoxicity and nephrotoxicity (Ali, 2003; Ali et al., 2011). In this study, renal damage was induced with gentamicin that had been administered to the rats at toxic doses for 7 days, and then the effect of *C. album* on any gastric ulcers and liver damage was analyzed. Several studies have also explored the therapeutic effect of *C. album* on liver and stomach ulcers (Baldi & Choudhary, 2013; Nigam & Paarakh, 2011). *Chenopodium album* seeds are also used in traditional medicine to strengthen the urinary tract wall (Hussain et al., 2022) These pharmacological properties support the results of this study. The urea and creatinine values of the G + CA group dropped significantly compared to the G group. Three other papers demonstrated that creatinine and urea levels in the blood increased upon gentamicin‐induced renal damage (Akyüz et al., 2021; Ulutaş et al., 2006; Üstebay et al., 2019), which is compatible with the present study. The G + CA group also exhibited a milder hyaline cast compared to the G group histopathologically. In other words, *C. album* had a therapeutic impact on renal damage – a first in the literature.

The combination of gentamicin and *C. album* exhibited alterations in relation to various biochemical markers, including creatinine, urea, total protein, glucose, potassium, and sodium. *Chenopodium album* is a good functional nutrient with many medicinal properties. With its concentration‐based increase in antibacterial and antioxidant properties, as well as its protective effects against nephrotoxicity, this plant has the potential to become a valuable therapeutic agent for a wide range of disorders.

## AUTHOR CONTRIBUTIONS

**Perihan Akbaş:** Conceptualization (lead); formal analysis (lead); investigation (lead); methodology (lead); project administration (lead); resources (lead); supervision (lead); validation (lead); writing – original draft (equal); writing – review and editing (equal). **Elife Kaya:** Conceptualization (equal); data curation (equal); investigation (equal); methodology (equal); writing – original draft (equal); writing – review and editing (equal). **Mustafa Makav:** Conceptualization (equal); data curation (equal); investigation (equal); methodology (equal); writing – original draft (equal). **Gülden Yıldız:** Data curation (equal); investigation (equal); writing – original draft (equal).

## CONFLICT OF INTEREST STATEMENT

The authors declare no conflicts of interest.

## ETHICS STATEMENT

This study was approved by Kafkas University Experimental Animal Research Ethics Committee (KAUHADYEK 2021/12).

## Data Availability

Despite the fact that appropriate data have been supplied in the form of tables and figures, the authors declare that if additional data are requested, they will provide them on a request basis.
